# Tinnitus: at a crossroad between phantom perception and sleep

**DOI:** 10.1093/braincomms/fcac089

**Published:** 2022-04-05

**Authors:** Linus Milinski, Fernando R. Nodal, Vladyslav V. Vyazovskiy, Victoria M. Bajo

**Affiliations:** Department of Physiology, Anatomy and Genetics, University of Oxford, Sherrington Building, Parks Road, Oxford OX1 3PT, UK

**Keywords:** phantom percepts, sleep, tinnitus, spontaneous brain activity, neural plasticity

## Abstract

Sensory disconnection from the environment is a hallmark of sleep and is crucial for sleep maintenance. It remains unclear, however, whether internally generated percepts—phantom percepts—may overcome such disconnection and, in turn, how sleep and its effect on sensory processing and brain plasticity may affect the function of the specific neural networks underlying such phenomena. A major hurdle in addressing this relationship is the methodological difficulty to study sensory phantoms, due to their subjective nature and lack of control over the parameters or neural activity underlying that percept. Here, we explore the most prevalent phantom percept, subjective tinnitus—or tinnitus for short—as a model to investigate this. Tinnitus is the permanent perception of a sound with no identifiable corresponding acoustic source. This review offers a novel perspective on the functional interaction between brain activity across the sleep–wake cycle and tinnitus. We discuss characteristic features of brain activity during tinnitus in the awake and the sleeping brain and explore its effect on sleep functions and homeostasis. We ask whether local changes in cortical activity in tinnitus may overcome sensory disconnection and prevent the occurrence of global restorative sleep and, in turn, how accumulating sleep pressure may temporarily alleviate the persistence of a phantom sound. Beyond an acute interaction between sleep and neural activity, we discuss how the effects of sleep on brain plasticity may contribute to aberrant neural circuit activity and promote tinnitus consolidation. Tinnitus represents a unique window into understanding the role of sleep in sensory processing. Clarification of the underlying relationship may offer novel insights into therapeutic interventions in tinnitus management.

## Teaser

Milinski *et al*. discuss the relationship between phantom sounds, sleep and sensory disconnection by synthesizing progress in tinnitus and sleep research. They propose a functional interaction between tinnitus-aberrant brain activity and natural brain state dynamics, with implications for tinnitus research, diagnostics, and potential for therapeutic interventions.

## Graphical Abstract

**Graphical Abstract:**
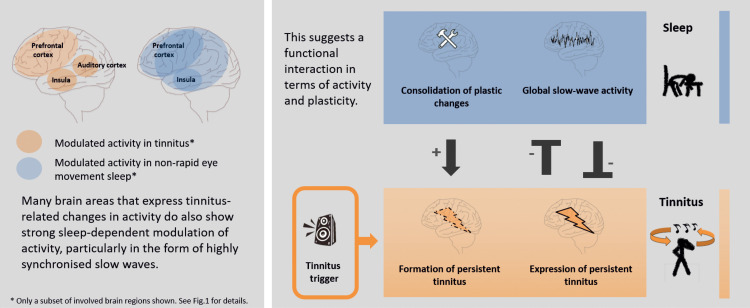

## Introduction

When we fall asleep, the main drivers of cortical activity change from being determined by external stimuli to being shaped mostly by internal dynamics, a process whose biological origin and functional significance is still not entirely understood. This drastic shift in vigilance state holds the potential to understand the generation of sensations likewise driven by internal processes. Phantom percepts and hallucinations, reported across all sensory modalities, are associated with brain activity patterns largely unrelated to external stimulation.^1,2^ However, the interplay between the neural activity underlying phantom percepts and the changes in spontaneous brain activity observed during the sleep–wake cycle has received little attention.

Subjective or primary tinnitus—hereafter referred to as tinnitus—is the most common phantom percept affecting at least 15% of the world population.^3,4^ It is defined as a perception of a continuous sound, usually taking the form of hissing or ringing with no identifiable corresponding acoustic source.^5^ According to the latest proposal in the field, tinnitus is defined as the conscious awareness of a tonal or composite noise for which there is no identifiable corresponding external acoustic source, which becomes tinnitus disorder when associated with emotional distress, cognitive dysfunction, and/or autonomic arousal, leading to behavioural changes and functional disability.^6^ Tinnitus can be transient, only lasting minutes, or persist for months to years and is commonly associated with distress, anxiety and sleep disruptions. To date, there is no available cure for tinnitus. Yet, our growing understanding of the condition and its prevalence makes it a promising model to investigate the interplay of sensory phantoms with sleep. Further, the auditory modality is commonly used to investigate sleep by means of assessing arousal thresholds to sound stimuli^7,8^ making an auditory phantom percept such as tinnitus a good model to investigate parallels between external and internal triggers of arousal.

In the last years, important advances have been made in identifying the neural correlates of tinnitus. Identified correlates include increased excitability and spontaneous activity along the auditory pathway, which can remain local or, especially in long-lasting tinnitus, encompass widely distributed brain regions^9–14^—areas where activity is highly sensitive to shifts in vigilance states across sleep and wakefulness (Box 1). This spatial overlap between areas affected by tinnitus and those involved in the change in vigilance state may lead to competition between pathological and natural drives for particular network activity. If pathological activity persists across vigilance states, tinnitus-related abnormal brain activity may result in a state of hyperarousal as is typical in some forms of insomnia and parasomnias^15–17^ where the emergence of local and global activation prevents sleep onset or interferes with natural sleep–wake dynamics (Box 2). Yet, importantly, internal drives for a shift in brain state have a remarkable ability to modulate local and global activity and may, in turn, interfere with local tinnitus correlates. This could impose a dynamic modulation of the phantom sound across the sleep–wake cycle, depending on the relative weight of homeostatic and circadian drives.

Box 1
**Neural activity and plasticity in tinnitus**

Hearing loss and auditory neuropathy (synaptopathy)^18,19^ without auditory threshold changes (hidden hearing loss)^20^ lead to reduced output from the cochlea and are at the basis of the most widely considered models for tinnitus generation.^21^ Neural plasticity plays a role in the pathogenesis of tinnitus.^22^ Typically, this involves a compensatory increase in neuronal gain to maintain homeostasis after the loss of afferent input, such as a decrease of inhibition and/or an increase of excitation, whereas gain modulation can be based on a variety of mechanisms such as cortical map plasticity, or changes in excitability and synchrony or spatiotemporal brain activity.^23^

Along the auditory brain, neural fingerprints of tinnitus in animal models are an increase in spontaneous activity and neuronal synchrony combined with changes in neural burst activity (dorsal cochlear nucleus;^24–27^ inferior colliculus;^28,29^ medial geniculate body;^30^ auditory cortex).^31–34^

The representation of distressing tinnitus goes beyond the auditory system, such as in the prefrontal cortex, the hippocampus and the amygdala.^35^ De Ridder *et al*.^36,37^ argue that tinnitus is only perceived once ‘salience’ brain networks, consisting of frontal and parietal areas, are involved in its representation. Changes are particularly observed in the limbic system, related to the emotional impact of a phantom sound and the formation of chronic tinnitus^10,36,38^ with neuronal hyperactivity in the NAcs^16^ or a reduction of amygdala activation by unpleasant sounds, possibly due to an ‘internal modification of emotional response’.^39^ Rauschecker^40^ proposed a malfunctioning corticostriatal gating system for sensory systems as a basis for tinnitus, involving NAcs and the ventromedial prefrontal cortex.

Box 2
**Sleep dynamics and local regulation**

Sleep and wakefulness dynamics are regulated by circadian and homeostatic factors, as outlined in the two-process model by Borbély.^62^ Sleep onset is marked by a progressive increase in cortical slow waves, the hallmark of NREM sleep and commonly measured as EEG delta activity (1–4 Hz). This activity is a marker for sleep of high intensity used as a proxy for differentiating between sleep stages,^63^ and it is a direct indicator for homeostatic sleep pressure. NREM sleep alternates with shorter phases of REM sleep during which a strong theta activity (6–9 Hz) is expressed in widespread brain areas and coupled with elevated gamma activity (<40 Hz).^63–65^

**Figure 1:**
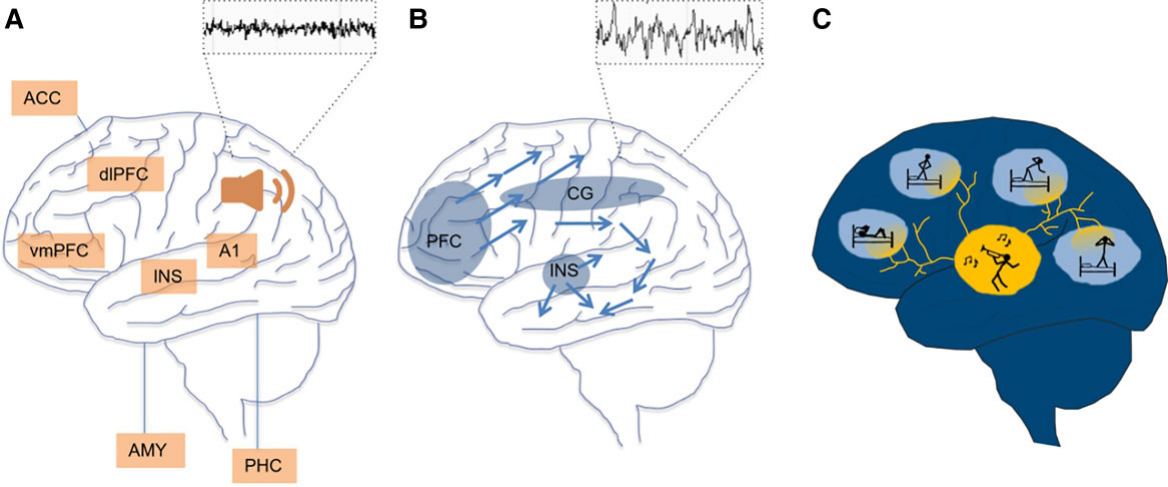
**Topographic overlap of brain areas involved in tinnitus and NREM sleep.** (**A**) Central brain regions where functional changes have been described in tinnitus during wakefulness, as depicted by the inlet showing exemplary wake EEG (figure adapted from).^35^ (**B**) Hubs and trajectories of SWA (as depicted by the inlet) during NREM sleep. Under high sleep pressure, NREM SWA is most pronounced in frontal areas,^49^ with spontaneous slow waves also originating in the INS and CG.^50^ While most SWA is local, it can propagate over large distances, mostly from medial prefrontal cortex to the medial temporal lobe or hippocampus^51^ and is therefore present in most regions that show functional changes in tinnitus. (**C**) Graphical depiction of tinnitus as ‘local wakefulness’ in the sleeping brain. If tinnitus-related activity persists during sleep, it may locally interfere with the expression of SWA in the affected areas, resulting in local wakefulness. This, in turn, may hinder the brain from entering global, restorative sleep. ACC, anterior cingulate cortex; AMY, amygdala; A1 primary auditory cortex; CG, cingulate gyrus; dlPFC, dorsolateral prefrontal cortex; EEG, electroencephalogram; INS, insula; PHC, parahippocampus; vmPFC, ventromedial prefrontal cortex.

While sleep is considered to be a global state, it is not uniformly expressed across the brain,^49^ a phenomenon commonly described as ‘local sleep’.^66,67^ Both, motor^68^ and sensory areas^69,70^ can show local modulations of SWA during sleep. SWA seems to gradually increase as a function of the previous wake duration and mostly in those brain areas that were involved in executing demanding tasks,^51,68,71^ although the topography of SWA is also shaped by the natural propagation trajectories of slow waves (Fig. 1B).^49–51^ The accumulation of sleep pressure depends on the time spent awake as well as on waking experience,^62,72,73^ as does the drive for localized sleep expression.^51,68,71,74^

Despite the local regulation of sleep, there is a natural drive for globally synchronized slow waves and off-phases in neuronal firing at the onset of overt sleep at high levels of sleep pressure. This synchronized activity can be necessary for the restorative function of sleep, whereas any local deviation, such as one region displaying wake-like activity, is likely to impair this.^61^

The significance of the aberrant activity observed in tinnitus depends on the formation and long-term maintenance of underlying circuitry, important factors in sustaining a phantom sound after an initial trigger. It is thought that neural plasticity following a triggering event plays a key role in tinnitus persistence (Box 1). Given the well-known role of sleep in mechanisms underlying synaptic plasticity and long-term consolidation of memory traces,^41,42^ it is important to consider how sleep may contribute to the cortical reorganization in tinnitus development, such as subsequent to reduction of sensory input.^43–45^

Here, we provide a critical overview and synthesis of the literature addressing the bidirectional interaction between tinnitus and sleep. We integrate our existing knowledge on tinnitus formation and its neurophysiological mechanisms with the current understanding of local and global sleep regulation in normal physiology and disease. Furthering the understanding of the link between tinnitus and sleep will have important implications for unravelling the effect of internal activities on brain-wide dynamics and offer new opportunities for the prevention and treatment of related pathologies.

## Tinnitus in the awake brain

The majority of evidence for the neural correlates of tinnitus and its symptoms stems from animal models and human patients assessed under general anaesthesia or during the waking state. Yet, it is important to acknowledge that these correlates encompass changes in both the auditory periphery and central cortical areas that are sensitive to natural brain state dynamics across sleep and wakefulness.

Tinnitus is most commonly associated with age-related hearing loss, termed presbyacusis, or an insult to the auditory system, such as noise exposure.^46^ Generally, many of the neural correlates of tinnitus have been linked to a partial loss of sensory input at the level of the cochlea or auditory nerve, even in patients with normal audiometric functions in what is known as hidden hearing loss^18,19^ (Box 1). Multiple functional changes along the auditory pathway have been associated with tinnitus. Among the most prominent are altered or increased spontaneous neural activity and network synchrony.

Supporting the model of tinnitus as a brain-wide phenomenon, functional connectivity studies established that non-auditory cortical areas are involved in generating the phantom percept, its chronification and associated distress^10,11^ (Box 1). For example, activity in the prefrontal cortex has been correlated with subjective tinnitus loudness.^47^ Furthermore, changes in cortical oscillations in tinnitus patients are not restricted to the auditory cortex but have also been observed in temporal, parietal and limbic cortices,^14^ as well as within the default mode network, the cerebellum and insula.^13^ While the pathophysiological significance of these changes is not entirely clear, a reduced resting-state connectivity between auditory and non-auditory regions has been associated with reduced phantom noise saliency.^48^ Such local and brain-wide changes in oscillatory brain activity associated with tinnitus can be relevant for tinnitus emergence and persistence and pose a vast area of interference with natural brain-wide dynamics (Fig. 1A).

Oscillatory brain activity, especially in the gamma-band frequency range, has long been thought to play a role in binding visual stimuli features into a conscious percept.^52^ Similarly, gamma-band activity could be related to the generation of pathological phantom percepts^53^ and tinnitus specifically.^53–55^ Interestingly, oscillatory coupling within the gamma frequency range is most widely distributed across the cortical hemispheres, including the prefrontal cortex, the orbitofrontal cortex and the parieto-occipital region, in patients with the longest tinnitus history.^9^ Furthermore, high-frequency activity in tinnitus has been described in the auditory cortex^54^ and has been related to hyperactivity in the regions in close vicinity to the deafferented cortical areas. This has been described to occur subsequent to hearing loss due to the loss of inhibition from deafferented regions, a phenomenon described as ‘the edge effect’.^56^ These findings highlight the importance of brain-wide oscillatory changes in persistent tinnitus^57^ and raise the question of how those changes in activity interact with normal dynamics of local and global oscillatory activity.

Spatiotemporal patterns of neuronal activity change markedly with attentional shifts,^58^ and, interestingly, training to shift attention away from the tinnitus percept by diversion or by integrating the phantom percept with multisensory stimuli can be used to alleviate it.^59^ Yet the arguably most drastic shifts in natural brain state occur as part of the sleep–wake cycle^60,61^ (Box 2). Many brain areas that express tinnitus-related changes in activity during the waking state do show strong sleep-dependent modulation of activity, particularly in the form of highly synchronized slow waves (Fig. 1A and B). To date, it remains an open question how this spatial overlap may affect tinnitus during sleep.

## Tinnitus in the sleeping brain

There are two perspectives on the interaction between tinnitus and sleep-dependent activity in overlapping areas that we are considering. First, the changes in network excitability associated with tinnitus may cause a state of hyperarousal. Given the extensive interconnectivity across many brain areas affected by tinnitus, even the emergence of spatially restricted cortical ‘arousal’ might result in a wide-reaching disruption of neural activity.

Localized cortical arousals are typical in parasomnias^75,76^—states of pathological co-expression of sleep in frontal brain areas and wake-like brain activity in motor and limbic regions.^16^ Such arousal disorders are associated with daytime sleepiness, anxiety and depression,^16,77^ a remarkable similarity to the typical comorbidities of tinnitus.^78,79^ In tinnitus, many brain regions show activation during the waking state^35^ with marked involvement of the limbic system^10,36,38^ such as neuronal hyperactivity in the nucleus accumbens (NAcs).^11^ While it has not been investigated whether such aberrant activity persists during sleep, it may produce alterations in global brain activity with parallels to a dissociated state. In fact, a recent study reported a high prevalence of parasomnia, sleep terrors, in tinnitus patients.^80^ It is of note here, that an important risk factor for parasomnias is sleep fragmentation,^81^ which can be caused by stress—a typical comorbidity of tinnitus—or possibly by the tinnitus itself (see section ‘Sleep architecture in tinnitus’ below). Yet, it remains to be investigated whether tinnitus can predispose for sleep terrors, somnambulism (sleep walking) or confused arousal.

Supporting the notion of auditory phantoms as arousal disorders there is a prominent example of a phantom perception strongly associated with arousal from sleep: the exploding head syndrome (EHS). In EHS, a sudden, high-intensity phantom sound is experienced during the transition into sleep, triggering awakening.^82,83^ EHS has been described as a parasomnia where dysfunction of various areas in the brainstem reticular formation can delay the general cortical switch-off when entering the sleep state. Here, some cortical areas show persistent arousal when other cortical areas have already transitioned into sleep.^83^ Arguably, if aberrant brain activity due to tinnitus persists in subcortical and cortical structures during the sleep state, it might cause disruptions in a similar way.

Another angle of interference between tinnitus and the sleeping brain may be illustrated by objective sensory stimulation with perceptual similarities to the subjective phantom perception. Sleep is traditionally defined as a state of relative sensory and global disconnection, likely taking place at the level of central brain areas.^84^ Yet, it is well known that the brain is highly receptive to auditory stimulation during sleep.^85^ Auditory evoked potentials are largely preserved up to the level of the auditory cortex^86,87^ and rhythmic stimulation can affect memory formation during sleep^88^ and modulate cortical slow-wave activity (SWA).^85^ Further, at a sufficient intensity level, auditory stimuli are potent triggers for suppressing non-rapid eye movement (NREM) SWA^89^ and initiating global arousal.^7,8,90,91^ Even continuous white noise can be detrimental to sleep^92^ and, specifically, lead to lighter sleep and increased arousals.^93^ This raises the question of whether a phantom noise would have the same effect, especially since tinnitus involves the spontaneous activation of auditory pathways (Box 1).

Tinnitus might therefore interfere with sleep at two levels: (i) As a form of local, dissociated awakening (Fig. 1C) where tinnitus-related changes in central oscillatory activity compete with fundamental functional hallmarks of sleep and (ii) as an arousal trigger equivalent to auditory stimulation in terms of both subjective experience and the activation of auditory pathways.

### Sleep architecture in tinnitus

If tinnitus interferes with sleep, this should be reflected qualitatively or quantitatively in the structure of the sleep–wake cycle—a notion that gained increased traction in recent years. Insomnia is indeed common comorbidity of tinnitus^94^ and sleep architecture assessed by polysomnography is remarkably similar between tinnitus patients with sleep complaints and primary insomnia patients.^95,96^ Studies where brain activity in tinnitus patients was compared to healthy controls highlight specific sleep alterations: Hébert *et al*.^97^ found subjectively reported sleep problems and reduced average EEG spectral power in the delta frequency band in tinnitus patients. Other studies identified a bias of tinnitus patients towards longer sleep latency^95^ and lighter sleep,^95,98^ correlated with subjective tinnitus loudness.^98^ It is worth noting that while sleep was consistently reported as shallower and less stable in tinnitus patients than in controls, tinnitus sufferers still entered all sleep stages, including slow-wave and REM sleep, indicating a certain robustness of sleep expression in the face of aberrant brain activity. It is also of note that the tendency towards lighter NREM sleep observed in tinnitus patients is similar to the effects observed in continuous white noise stimulation.^93^

Further correlational findings support the possibility that tinnitus can change global sleep architecture. Alster *et al*.^99^ assessed patients with long-lasting (>6 months) tinnitus to find that 77% had increased delayed sleep, early morning awakenings, mid-sleep awakenings, morning fatigue, and chronic fatigue as compared with healthy controls. Similarly, more recent studies based on patient ratings demonstrate a correlation between subjective tinnitus loudness and symptoms of changed sleep, such as sleep disruptions^100–104^ and the ability to initiate sleep.^104^

Interestingly, the magnitude of sleep disturbance can change during the temporal progression of tinnitus. Folmer and Griest^105^ described that the subjective ratings of tinnitus severity and sleep disruptions were lower 1 year or 22 months after the initial assessment. This effect could be explained by treatments implemented by participants in this study, such as psychological counselling, stress management, or the use of medications to improve sleep.^101^ Other studies, without regulated treatment, report stable or worsening tinnitus over time,^106,107^ although Scott et al.^106^ found, in parallel, improved tolerance over time, which in itself can be beneficial to sleep. Benefits on sleep have also been reported in tinnitus patients who received auditory stimulation^108^ or made use of muscle relaxation techniques.^109^ While strategies such as relaxation techniques might help sleep in their own right, the renormalization of limbic activity in tinnitus patients^10,11^ and therefore reduction of aberrant activity that may directly underly sleep disruption could represent a promising target for therapeutic intervention.

It is important to acknowledge that the association between tinnitus, altered brain activity, and altered sleep architecture, while robust, is so far correlational in nature. It has been suggested that the sleep disruptions in tinnitus could be explained by hyperarousal, a condition that makes patients prone to both tinnitus distress and insomnia.^110^ However, while not excluding this possibility, the mechanistic overlap between sleep dynamics and tinnitus-related brain activity uncovered in recent years now offers sufficient grounds for considering a direct interaction. Tinnitus is a brain-wide phenomenon (Box 1), encompassing persistent changes in brain activity and circuitry in frontal, parietal and limbic regions,^9–11,13,14,35^ areas directly involved in sleep expression (Fig. 1)^49–51^ and homeostatic sleep regulation.^111,112^ This highlights a general vulnerability of sleep to tinnitus-related activity.

At the same time, the spatial overlap between sleep- and tinnitus-dependent brain activity has a further implication. Theoretically, any drive to shift brain activity from the waking to the sleep state may act in conflict with the drive to maintain aberrant brain activity in those regions. The question remains, therefore, whether the tendency of affected neuronal populations to engage in stereotypical firing during sleep could temporarily change tinnitus-associated activity patterns and possibly affect tinnitus generation dependent on natural brain state dynamics.

### Sleep homeostasis and tinnitus

The arguably most prominent dynamic of the functional brain state is the reversible change from alert wakefulness (high-frequency, desynchronized activity) towards NREM sleep. A hallmark is the emergence of widespread, synchronized cortical SWA (Box 2). Globally synchronized SWA is often considered to be an important factor in mediating recovery functions of sleep—from synaptic renormalization to metabolic restoration.^61,113^ SWA is especially high during early sleep after an extended period of active wakefulness,^72,74,114^ and arousal thresholds are typically highest during intense sleep, characterized by pronounced SWA. Sensory thresholds gradually increase with high-intensity sleep^8^ and disconnection correlates with the magnitude of SWA^63^ and the phase of coordinated oscillation of the sleep spindle band.^115^ While it remains to be determined whether the primary biological role of sensory disconnection during sleep is to protect spontaneous brain activity, it is likely essential in this respect. An emergence of aberrant patterns of brain activity during tinnitus^9–11,13,14,28–27^ is expected to prevent globally coordinated activity such as SWA and decrease the global arousal threshold, which will therefore interfere with the normal dynamics of sleep homeostasis,^62^ and may impair the restorative functions of sleep.

The evolved drive to express sleep-dependent brain activity even in the face of local disruptions, such as under very high sleep pressure, might, however, mitigate the effect of tinnitus on spatiotemporal brain activity. Entrainment of local cell populations with cortical SWA is likely based on both intrinsic and extrinsic factors. As for the former, evidence suggests that elevated spiking and synaptic activity, typical for the awake state,^112^ results in increased metabolic load and accumulated need for cellular maintenance.^61^ To offset wake-dependent changes and restore cellular homeostasis, neurons may enter periods of silence, so-called off periods, which are known to increase after sleep deprivation.^112,116,117^ The extensive cortical interconnectivity promotes global synchronization of SWA, driving more cells towards an on-off firing pattern^61,118^—an extrinsic drive for individual neurons to entrain with global SWA. In addition, the occurrence of off periods may contribute to sensory disconnection by interrupting the propagation of information across cortical areas^119^ and thus shunt the effect of aberrant activity on widespread cortical networks.

**Figure 2:**
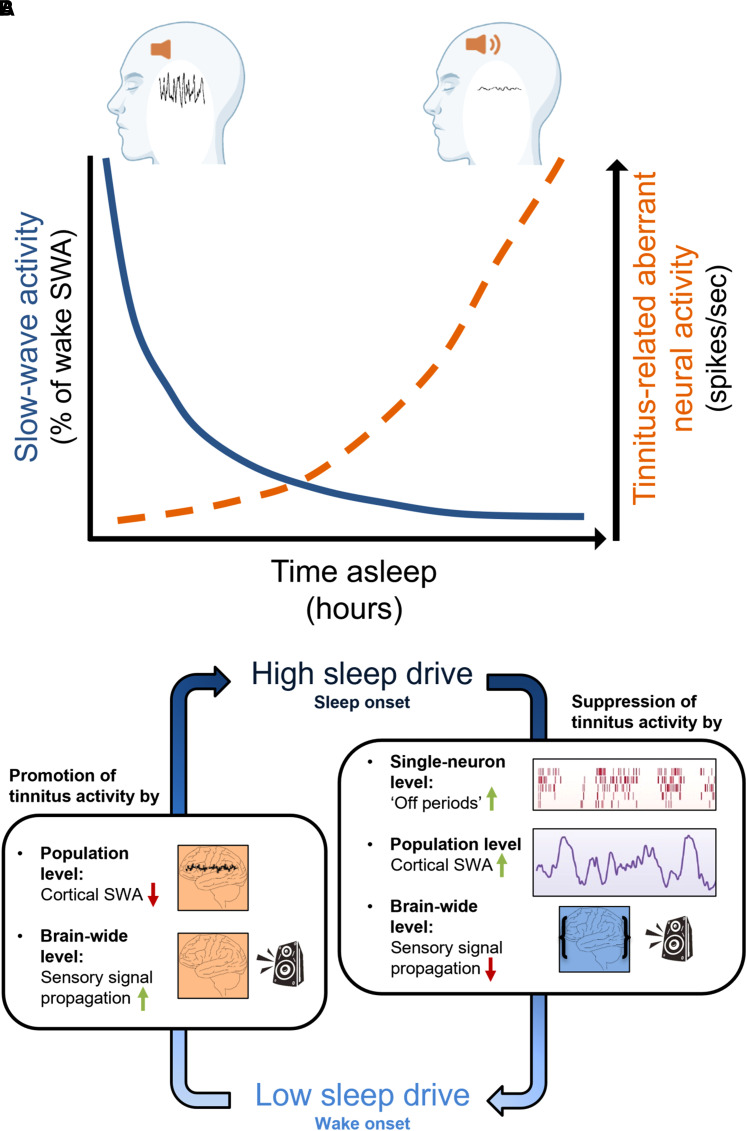
**Interaction between tinnitus and sleep.** (**A**) Proposed time course of tinnitus modulation during sleep. When homeostatic sleep pressure is high, global SWA can override local aberrant activity and may reduce the impact of tinnitus-related brain activity. As time asleep increases, sleep pressure dissipates and the magnitude and synchrony of SWA decreases, allowing aberrant brain activity to interfere with and eventually disrupt overt sleep. (**B**) Mechanisms for the interaction between tinnitus and sleep shown in (**A**). During NREM sleep under high sleep pressure (right hand side) global activity could supress local deviations in brain activity present in tinnitus via: (i) the intrinsic cellular drive to enter an ‘on-off’ firing pattern, which is further promoted by (ii) entrainment with local and global cortical SWA, which may disrupt local tinnitus-related activity and (iii) sensory decoupling during sleep, which may reduce the contribution of tinnitus-related activity in the peripheral auditory system to the perception of tinnitus. During wakefulness under low sleep pressure (left hand side), the drive for SWA is minimal and signal propagation unhindered, which may ultimately promote tinnitus saliency.

An intriguing possibility is that these factors, entraining with cortical SWA and reduced signal propagation, could disrupt abnormal tinnitus-related activity, resulting in a suppression of phantom sound generation or its perception. Thus, physiologically elevated sleep pressure, associated with high global SWA, might ‘override’ aberrant tinnitus-related cortical activity, or suppress its effect on wider brain networks (Fig. 2A) and allow for the expression of deep NREM sleep under such conditions. In fact, deep NREM sleep (sleep stages 3 and 4, and slow-wave sleep) is evident in tinnitus patients even when they otherwise show sleep disruptions or changed sleep architecture.^95,97,98^ Further, patients have been reported to describe sleep as a state of experiencing reduced tinnitus.^37,107^ Elevated sleep pressure could temporarily prevent local or global arousal and suppress the perception of a phantom sound. In turn, as the propensity for pronounced SWA decreases during sleep, along with the decrease in arousal threshold, aberrant brain activity is expected to regain saliency and the potential for sleep disruption.

Therefore, tinnitus may interfere with sleep in a sleep-stage or time of the night-dependent manner. Specifically, while tinnitus may be dampened during initial high-intensity sleep after a period of consolidated wakefulness, this effect is expected to gradually decrease once sleep pressure alleviates and cortical SWA becomes less synchronized. According to this hypothesis, tinnitus patients would miss out mostly on early-morning sleep when sleep pressure is relatively low and experience high tinnitus loudness in the early phase of the wake period or when sleep is disrupted (Fig. 2B). Experimental assessment of this possibility would require a comparison of sleep parameters between early and late sleep periods, ideally, while tracking an objective neural correlate of tinnitus such as local hyperactivity^11,33,34^ and its effect on wider brain activity during the time course of consolidated sleep.

While tinnitus loudness is modulated by factors such as stress^120^ and learned disengagement^59^ and does not always present a clear daily trend during waking hours,^121,122^ there is evidence that tinnitus loudness is often highest in the morning,^123,124^ anecdotally reported as ‘morning roar’ by tinnitus patients.^124^ Interestingly, Nicolas-Puel *et al*.^123^ found an increase in tinnitus loudness after periods of sleep in patients who reported fluctuating tinnitus saliency, yet patients with constant saliency were also described, suggesting that the magnitude of the effect of sleep homeostasis on tinnitus might vary depending on the individual type of tinnitus suffered by the patient. Importantly, the proposed hypothesis suggests that tinnitus-related brain activity may be suppressed during global sleep and while the drive to maintain this state is maximal, i.e. during initial sleep after an extended waking period. While patients do retrospectively describe sleep as a state of reduced tinnitus,^37,107^ it remains to be addressed how tinnitus saliency is modulated during the course of global sleep.

SWA during sleep is regulated not only on a global but also on a local scale, and local slow waves can be dissociated from the global state of vigilance^66,68,74^ (Box 2). This raises the possibility that a shunting effect of sleep on tinnitus may be further modulated by factors affecting local sleep regulation, such as the type and duration of waking experience.^68,74^ The notion that local sleep may interfere with network function or sensory perception^71^ may open up a novel therapeutic opportunity for tinnitus prevention or treatment. Specifically, local induction of slow waves in the auditory cortex using auditory clicks or sleep deprivation protocols^125,126^ may prevent the emergence or propagation of aberrant neural activity, which could improve sleep quality.

The possibility that sleep disturbance in tinnitus and in primary sleep disorders share some of the same local mechanisms underlying the alteration of global activity remains to be addressed. Investigating this possible commonality would lead to further understanding of the functional tinnitus correlate and could inform common treatment directions.

## Sleep in tinnitus pathogenesis

The functional overlap between sleep dynamics and tinnitus expression may not only govern acute effects on tinnitus saliency but also play a role in state-dependent plasticity of affected brain areas. The pathogenesis of tinnitus consists of two stages: First, the initial triggering of the tinnitus, such as an insult to the auditory periphery, and second, a process that promotes the formation of a persistent phantom sound.^127^ Shore and Wu^22^ argued that cochlear synaptopathy—the loss of cochlear synapses—is necessary but not sufficient for tinnitus formation and that additional plasticity processes are required. These entail changes in neuronal gain to maintain homeostasis after the loss of afferent input, such as a decrease of inhibition and/or an increase of excitation. Gain modulation can be based on a variety of mechanisms such as cortical map plasticity, changes in excitability and synchrony or spatiotemporal brain activity.^23^ Tinnitus also involves consolidation of long-range connectivity such as between the auditory and the limbic system^10^ or the functional coupling across cortical hemispheres, including prefrontal cortex, orbitofrontal cortex and the parieto-occipital region.^9^ Sleep, being a functional brain state central to mediating plasticity,^41,42,128–131^ might play an important role in consolidating such changes.

### Cortical map plasticity

An example of early plasticity observed in tinnitus is the reorganization of cortical tonotopic maps.^31,46,132^ The receptive fields of cortical neurons are dynamic entities, and the spatial arrangement in the form of ‘cortical maps’ can change drastically in response to altered afferent signals.^133^ Cortical remapping after sensory deprivation has been described in the somatosensory,^134^ visual^135^ and auditory system.^136,137^ Following cochlear lesions^138,139^ or noise exposure,^132,140^ the cortical areas with reduced input from the affected auditory periphery change the frequency tuning of their neurons towards the closest non-affected frequency representations. This, in turn, increases the representation of those edge frequencies and leads to higher synchronous gain,^141^ which is itself a functional tinnitus correlate.

Tinnitus has indeed been correlated with cortical map reorganization.^46^ Triggering renormalization of cortical maps can improve tinnitus in animals^31^ and humans.^142^ Although the exact relevance of cortical map plasticity in tinnitus formation is still debated,^143^ it could be a driver in early tinnitus pathogenesis, where sleep is likely to play a role. State dependence of cortical map plasticity has been demonstrated in the visual system following ocular deprivation.^135^ Specifically, the lack of visual afferent input leads to the reorganization of cortical maps in the form of ocular-dominance plasticity, which is promoted by sleep and inhibited by sleep deprivation.^128^ Frank *et al*.^44,129^ showed that particularly NREM sleep drives cortical reorganization after monocular visual deprivation. In fact, the amount of NREM sleep has been found to correlate with the degree of ocular-dominance plasticity.^129^

Cortical map reorganization after the loss of cochlear afferents, a major trigger for hearing loss and tinnitus,^46^ shows remarkable similarity to the plasticity observed after ocular deprivation. It remains to be addressed whether such processes in the auditory modality are also state-dependent or even fundamentally driven by NREM sleep.

### System-level plasticity

Beyond plastic processes restricted to the auditory pathway, sleep may be involved in brain-wide changes associated with tinnitus pathogenesis^9,11,13,14,144,145^ (Box 1). Altered activity in non-auditory brain regions such as hyperactivity in the NAcs^38^ or reduced amygdala activation^39^ suggest that system-level plasticity plays a role in tinnitus pathogenesis,^145^ subsequent to changes in neuronal synchrony, burst activity and firing rates reported in the different processing centres along the auditory pathway^24,25,28,30,32,132^ (Box 1). The mechanism of transition from local plasticity in the auditory pathway towards brain-wide changes observed in persistent tinnitus remains to be shown. Yet, interestingly, it is known from studies investigating memory consolidation that plasticity observed across cortical regions can be driven by local functional changes—a process promoted by sleep.^146^ Several mechanisms are considered to be part of this sleep-dependent plasticity, including replaying memory traces^41^ or synaptic strengthening and weakening.^113^ On the level of spatiotemporal brain activity, phase locking of spindles and ripples during the upstate or the depolarized phase of the slow oscillation is thought to contribute to memory formation,^147^ while depolarization of local cell populations can increase the chance of concerted neural firing and, in turn, promote plasticity.^146^ This process, often termed memory consolidation, usually entails a shift of memory retrieval associated activity from the hippocampus to the neocortex subsequent to associative memory tasks.^130,131^

A similar sleep-dependent process could drive the gradual involvement of widespread brain areas in tinnitus. While the hippocampus can indeed show structural changes in tinnitus^148^ the possibility remains that aspects of sleep-dependent systems memory consolidation can be triggered by local changes in other areas, such as regions in the auditory pathway with the most pronounced aberrant activity in tinnitus.^27,28–26,32,33,132^ It remains to be investigated how such local changes are reflected in global brain plasticity, especially during a state prone to structural reorganization such as sleep.

**Figure 3:**
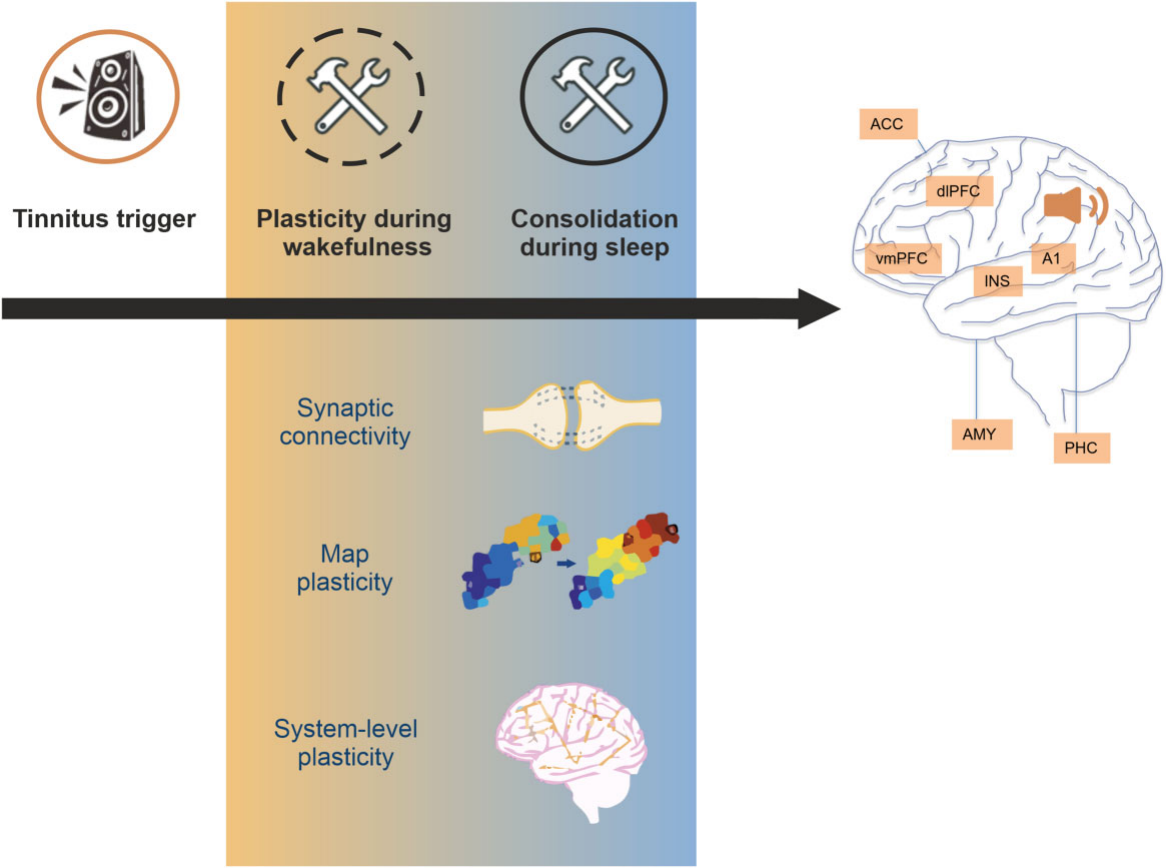
**Sleep-mediated plasticity may contribute to tinnitus development.** Following a tinnitus trigger (such as noise overexposure), neural plasticity is driving the development of tinnitus towards a brain-wide network representation as highlighted by central brain areas demonstrating altered connectivity or activity in tinnitus. This includes changes on the level of synaptic connectivity, cortical map reorganization (as depicted by a change in receptive field organization, heat maps adapted from)^154^ and systems-level plasticity affecting global connectivity—all processes directly affected or consolidated by the sleep process. The formation of persistent tinnitus may be fundamentally driven by sleep-dependent mechanisms.

Cortical remapping and systems-level plasticity are two examples of sleep-dependent plasticity that may help explain circuitry changes underlying the development of phantom percepts. Yet, the scope of sleep-mediated plasticity processes involved in tinnitus might be considerably wider, including, for instance, synaptic upscaling or downscaling^149,150^ or spatially restricted synapse plasticity^151^ (Fig. 3). Future investigations will need to explore these possibilities in the context of tinnitus research and treatment. Controlled sleep restriction paradigms are already used for the treatment of insomnia^125,126^ and guided plasticity, such as through auditory stimulation during sleep,^152,153^ could affect tinnitus chronification and possibly improve recovery. It should be noted that brain state dynamics and associated windows of plasticity present opportunities to capitalize on the brain’s natural processes for treating evasive conditions such as tinnitus.

## Conclusion

Sleep plays an essential role in health and wellbeing^155,156^ and sleep disturbances are present in most psychiatric illnesses.^157^ Therefore, it is surprising that sleep has received so little attention in the investigation of pathological alterations of brain activity associated with phantom percepts, especially when stimuli subjectively equivalent to the phantom are known to interfere with sleep. In fact, many of the comorbidities associated with phantom perceptions might be mediated through sleep alterations. In tinnitus, we argue that the functional overlap between networks generating the phantom percept and areas sensitive to sleep–wake dynamics offers a mechanism for interference that has, surprisingly, never been considered. The role of natural brain states in phantom percept salience and pathogenesis is largely unexplored. Further work is needed to understand this relationship. Ultimately, this will help to explain comorbidities, to reduce biases in diagnostics and to develop new treatments (Box 3).

Box 3
**Outlook: sleep in tinnitus research, diagnosis and treatment**

Given the likely impact of sleep–wake dynamics on tinnitus expression and development outlined in this article, we suggest a set of general considerations to account for these factors in tinnitus research and clinical practice.

- First, the proposed effect of sleep on tinnitus severity has important implications for tinnitus diagnosis. The main factors determining sleep–wake dynamics are the homeostatic sleep drive (based on sleep–wake history, see Box 2) but also circadian drives. Circadian rhythms and homeostatic sleep pressure might both contribute to the subjective tinnitus severity. Yet little attention is paid to these factors when testing for tinnitus in both animal models and human patients. Circadian rhythms have been observed in the cochlea^158^ and in the inferior colliculus,^159^ suggesting that the time of day is an important factor in auditory processing. To control for circadian impact, the diagnosis of tinnitus should not be restricted to a single assessment, but monitored across different times of the day. To take into account the possible role of sleep pressure at the time of assessment, patients could be routinely asked for their recent sleep–wake history. Integrating those measures into the standard regime of tinnitus diagnostics could lead to a more accurate assessment in the clinical context, and also reduce the marked variability that has been described across animal and human studies.^127^
- Second, the hypothesis laid out in this review suggests that sleep interferes with tinnitus expression. Exploring this relationship could offer important insights into the generation of the phantom sound based on brain activity and may offer a natural mechanism for tinnitus mitigation that could be harnessed for clinical treatment development. For example, sleep restriction protocols^125,126^ or manipulation of sleep-like brain activity (such as cortical slow waves)^88^ via auditory or electrical stimulation may hold potential for tinnitus mitigation.
- Third, the investigation of the possible role of sleep in mediating the time course of tinnitus development could highlight critical time windows relative to the trigger event (e.g. noise overexposure), where sleep promotion or prevention could reduce the likelihood of developing persistent tinnitus.

We outlined a framework for the interaction between sleep and tinnitus based on current evidence in the field of sleep and tinnitus research (see graphical abstract). We describe a functional overlap between brain areas affected by sleep–wake dynamics and those commonly involved in tinnitus and, possibly, in regulating its saliency. We conclude that tinnitus-related oscillatory dynamics and firing activity may compete, to a degree, with sleep–wake-related patterns of brain activity. We posit that tinnitus can lead to persistent local wakefulness and thus interfere with sleep onset and sleep maintenance. In turn, once the drive to express global sleep reaches a level sufficient to impose global SWA, this may suppress aberrant local activity and reduce cortical signal propagation, mitigating the tinnitus percept during sleep of high intensity. Factors that determine the sleep drive, primarily homoeostatic and circadian in nature, could modulate tinnitus saliency across the day. A second implication of the functional overlap between tinnitus and sleep relates to the role of sleep in mediating brain plasticity. We posit that mechanisms driving plastic changes in brain connectivity during sleep could promote brain-wide changes necessary for tinnitus maintenance. In summary:

- Sleep oscillatory activity is at odds with brain-wide functional changes observed in tinnitus.
- Tinnitus-related brain activity holds the potential to interfere with these dynamics, while deep NREM sleep may, in turn, suppress such activity temporarily.
- Sleep-dependent brain plasticity may consolidate changes in brain connectivity during tinnitus formation.

The proposed framework, while hypothetical, offers a new perspective to interpret existing results and guide future research. It may, further, help to acknowledge the interplay between normal and pathological brain activity affecting the perceptual state. Tinnitus is an example of a condition where clinical diagnostics and basic research are sufficiently advanced to tackle this relationship as a logical next step in the search for effective treatments. Finally, tinnitus offers a unique window into the more fundamental question of how sleep impacts perception and opens new perspectives for tackling the neurophysiological mechanisms of sensory disconnection during sleep.

## Data availability statement

Data sharing is not applicable to this article as no new data were created or analysed in this study.

## Abbreviations

ACC =: anterior cingulate cortex
AMY =: amygdala
A1 =: primary auditory cortex
CG =: cingulate gyrus
dlPFC =: dorsolateral prefrontal cortex
EEG =: electroencephalogram
EHS =: exploding head syndrome
INS =: insula
NAcs =: nucleus accumbens
NREM =: non-rapid eye movement
PHC =: parahippocampus
REM =: rapid eye movement
SWA =: slow-wave activity
vmPFC =: ventromedial prefrontal cortex
